# Effects of Kinesiology Tape on Quadriceps Muscle Strength in Female Futsal Players: A Longitudinal Pilot Randomized Controlled Trial

**DOI:** 10.3390/healthcare13233035

**Published:** 2025-11-24

**Authors:** Norah A. Alshehri, Sarah A. Alshehri, Ahmed M. Abdelsalam, Nadia M. I. M. Gouda, Abdulrahman M. Alshehri, Abdullah A. Alrasheed, Joud S. Almutairi, Dina S. Almunif, Khalid F. Alsadhan

**Affiliations:** 1 Department of Family and Community Medicine, College of Medicine, King Saud University (KSU), P.O. Box 2925, Riyadh 11461, Saudi Arabia; aalrasheed1@ksu.edu.sa (A.A.A.); jalmutairi1@ksu.edu.sa (J.S.A.); dalmunif@ksu.edu.sa (D.S.A.); kalsadhan@ksu.edu.sa (K.F.A.); 2 University Family Medicine Center, King Saud University Medical City, Riyadh 11495, Saudi Arabia; 3 College of Sport Sciences and Physical Activity, King Saud University (KSU), Riyadh 12371, Saudi Arabia; pt.saraalsh@hotmail.com; 4 Department of Biomechanics and Motor Behavior, College of Sport Sciences and Physical Activity, King Saud University (KSU), Riyadh 12371, Saudi Arabia; amohmed@ksu.edu.sa; 5 School of Technology and Environment, EIT Faculty, Nova Scotia Community College e-Campus, Halifax, NS B3K 2T3, Canada; nadia.gouda@nscc.ca; 6 Office of the Vice President for Projects, King Saud University (KSU), Riyadh 11451, Saudi Arabia; engalshehri@ksu.edu.sa

**Keywords:** kinesiology tape, quadriceps strength, female athletes, futsal, randomized controlled trial, sports performance

## Abstract

**Background/Objective:** Kinesiology tape (KT) is commonly used in sports medicine and rehabilitation, but its impact on muscle strength over time remains unclear. Female futsal athletes experience high quadriceps demands and are at risk of anterior cruciate ligament injury; however, this population remains understudied. The purpose of this study was to investigate the longitudinal effect of repeated kinesiology taping applications on quadriceps muscle strength and lower limb function in female futsal players. **Method:** A longitudinal pilot randomized controlled trial was conducted during the Saudi Universities Sports Federation Futsal Championship. Twelve female athletes (aged 19–25 years) were randomly allocated to a KT (*n* = 6) or control group (*n* = 6). The KT protocol followed the standardized quadriceps facilitation guidelines and was applied repeatedly over 30 days. We measured isometric strength (hand-held dynamometer), eccentric/concentric torque and power (Biodex System), and functional performance (single-leg hop). Nonparametric tests (Wilcoxon signed-rank and Mann–Whitney U) and mixed ANOVA were used for analysis. **Result:** Post-intervention, the KT group demonstrated significant improvements in isometric strength (*p* = 0.03, r = 0.90), eccentric/concentric strength (*p* = 0.03, r = 0.90), and lower limb function (*p* = 0.03, r = 0.90). The between-group comparisons showed significant advantages for the KT group in isometric (*p* = 0.01, r = 0.83) and eccentric/concentric strength (*p* < 0.05, r = 0.67–0.74), but not in lower limb function (*p* = 0.20, r = 0.37). **Conclusions:** Repeated kinesiology taping over a 30-day period led to statistically greater longitudinal improvements in quadriceps muscle strength but did not affect functional performance. Kinesiology taping represents a non-invasive, low-cost treatment option for quadriceps strength measures in sports characterized by higher demands on the quadriceps, especially for female athletes with contributing injury risks. Further trials with more participants and a longer follow-up should be conducted.

## 1. Introduction

Kinesiology tape (KT), developed by Kenzo Kase in the 1970s, has gained popularity in sports medicine and rehabilitation following exposure at the Beijing Olympics (2008) [1,2]. Survey data suggest that up to 67% of athletes are using KT for injury prevention and rehabilitation [3]. KT is elastic (skin-like) and unlike rigid taping. KT allows for external support and considers a person’s full range of motion [2].

A complex interaction of biomechanical and physiological mechanisms may influence musculoskeletal function. Elastic recoil from KT lifts the skin, potentially lessening pressure on the subcutaneous mechanoreceptors and increasing the amount of interstitial space; this may improve local blood circulation and lymphatic drainage [1]. The decompression effect may reduce pain and swelling following vigorous activity and injury. During the neuromuscular process, KT provides mechanical stimulation through the skin, and potentially increases central nervous system afferent feedback, motor unit recruitment and proprioception [2]. Biomechanically, KT provides gradual, corrective forces to the skin and fascia that may help realign the joint position and improve dynamic stability [2].

Quadriceps strength is vital to success for a futsal athlete, influencing sprinting with and without ball control and accuracy of goal kicking. Futsal requires rapidly shifting positions (acceleration, deceleration, and changes in direction), which ultimately places large strain on the knee joint and the quadriceps [4]. The likelihood of injury in futsal sports is higher than it is in football. This statement is consistent with Ruiz-Pérez et al.; in futsal, sports injuries are about 2.7 times more common than they are in soccer [4]. Regardless of the environment, female athletes are at a significantly greater risk of incurring knee injuries, including anterior cruciate ligament (ACL) ruptures. They are reported to have a 2–8 times higher chance of experiencing ACL injuries compared with males because of a combination of biomechanical and hormonal factors [5]. Even though KT’s physiological mechanisms are the same, the high quadriceps demand in futsal, combined with sex-specific injury risks, emphasizes the need to study its effects on female athletes.

While KT is very popular, the evidence on its effect on muscle strength is inconsistent. Other experimental studies have shown a 10–15% improvement in eccentric quadriceps peak torque in healthy women [6]. One meta-analysis provided a fairly strong or moderate pooled effect on lower limb strength after 48 h of KT use (SMD = 0.46, *p* < 0.0001) [7]. Another meta-analysis implied benefits for people with fatigued muscles and certain chronic musculoskeletal conditions, but none of the studies on healthy, uninjured athletes reported consistent results [8]. Some studies reported that KT could improve peak torque, but only when combined with either rehabilitation or exercise programs, and these results were context-specific and not replicated [9].

In contrast, many well-controlled trials have reported no effect. A randomized placebo-controlled trial (RCT) found healthy volleyball players had no significant difference in quadriceps electromyographic activity or flexibility [10]. Many of the soccer studies reported no changes in isometric knee extension force immediately or 24 h after KT application [11,12]. A blinded RCT found no consistent facilitation of quadriceps strength over 5 days of KT use on healthy individuals [13]. In female university students with hamstring tightness, KT resulted in an instantaneous enhancement in hamstring extensibility, but no significant change in quadriceps isometric strength [14]. Similarly, controlled crossover studies on healthy athletes found no important improvement in knee extensor strength [15]. More recent meta-analyses have reported these findings as heterogeneous and determined that the overall evidence for strength enhancement is weak and inconsistent [16,17]. Most studies have measured only acute or short-term outcomes by testing for strength immediately or between 24 and 48 h after KT application [11,12,14,15], whereas the present trial evaluated repeated applications over a 30-day period to assess longitudinal effects. The differences in study design, participant population, and practice context may help to explain these inconsistencies. These inconsistencies highlight the importance of purposeful trials that address population-specific characteristics in conjunction with sport-specific demands.

While KT has been widely researched, most trials have either targeted male athletes or sports other than futsal. The observational evidence suggests that futsal athletes use KT frequently as a part of injury prevention and recovery. However, no RCTs have investigated the long-term or longitudinal impact of repeated KT applications on quadriceps muscle strength in female futsal athletes, even given their higher risk of injury and dependence on quadriceps performance. This underlines a critical knowledge gap, particularly for female futsal players—an under-represented group facing high quadriceps demands and elevated injury risk. Given the rapid global growth of futsal and the injury burden among female athletes, addressing this gap carries both clinical and performance significance. To our knowledge, this is the first randomized controlled trial evaluating the effects of KT on quadriceps muscle strength in female futsal athletes.

Therefore, the aim of this trial was to explore the effects of repeated kinesiology tape applications over 30 days on isometric and eccentric/concentric quadriceps muscle strength in female futsal athletes, which was the primary outcome. The trial also assessed the change in lower limb function as the secondary outcome. It was hypothesized that longitudinal KT application would provide a more significant improvement in quadriceps muscle strength compared with control taping.

## 2. Materials and Methods

### 2.1. Aims and Research Questions

The aim of this longitudinal pilot randomized controlled trial (RCT) was to evaluate the effects of repeated kinesiology taping applications over a 30-day period on quadriceps muscle strength and lower limb function in female futsal players.

Primary Research Question:

Does repeated kinesiology taping improve quadriceps muscle strength, measured as isometric, concentric, and eccentric performance?

Secondary Research Question:

Does longitudinal kinesiology taping enhance lower limb functional performance more compared with not taping?

### 2.2. Study Design, Setting, and Participants

#### 2.2.1. Study Design

This study was a longitudinal pilot randomized controlled trial (RCT) with a parallel-group, single-blinded design. The assessor performing pre- and post-measurements was blinded to group allocation; however, participant blinding was not feasible because kinesiology taping is a visible intervention. Although participant expectation bias could not be completely eliminated, several measures were implemented to minimize its potential impact. Standardized verbal instructions and testing procedures were consistently applied to both groups during all strength and functional assessments.

We used a no-tape control group because the primary objectives of this pilot trial were to assess feasibility, estimate effect sizes, and observe longitudinal responses to repeated KT applications in female futsal players under real-world training and competition conditions.

The participants were randomly assigned to the experimental (kinesiology taping) or control group (no taping). Randomization was completed by an independent researcher using a computer-generated sequence in Microsoft Excel (version 365) to ensure allocation concealment. We assessed quadriceps strength and function before and after a 30-day repeated taping intervention using valid and reliable instruments (see Methods, Section 2.6).

#### 2.2.2. Study Setting

This study was conducted between January and September 2023 during the first Saudi Universities Sports Federation (SUSF) Futsal Championship, hosted by King Saud University. The players represented six universities across Saudi Arabia.

#### 2.2.3. Study Participants

The participants were purposively sampled from a target population of 93 players at the SUSF Futsal Championship for six universities across Saudi Arabia. In total, 12 female athletes were recruited from the total pool who met the inclusion criteria, and then randomly assigned either to the experimental or control group.

Inclusion criteria. The participants were included if they met the following criteria:

- Age: from 19 to 25 years of age.
- Body mass index (BMI): from 18.5 to 25 kg/m^2^.
- Practicing sports: The common practice of engaging in sports activity or general fitness training.
- Previous kinesiology taping: They had not previously applied any type of kinesiology tape on the thighs for the last six months before this study commenced. A six-month duration of cables was used in this study to not allow for any potential carry-over or adaptation effects occurring on their earlier (pre) performance [17].

Exclusion criteria. The participants were excluded if they met any of the following criteria:

- Medical history: Previous history of knee or back pain, lower limb surgeries, or any joint deformities.
- Contraindications: Allergy to kinesiology tape, open wounds, cancer, deep vein thrombosis, enlarged lymph nodes, diabetes, fragile skin, or pregnancy.
- Recent injuries: Muscular injuries in the lower limbs that had not been sustained in the last six months before this study.

### 2.3. Sample Size and Power Analysis

An a priori power analysis was performed in G*Power 3.1 for the Mann–Whitney U test (two-tailed, α = 0.05, power = 0.80), using a large, but conservative effect size (Cohen’s d ≈ 1.0), based on previous kinesiology taping research [18]. This indicated that a minimum of 16 participants would be required.

However, given the real-world constraints of recruiting competitive female futsal athletes during a national championship, 12 participants (6 per group) were enrolled. This sample size was considered for an exploratory, longitudinal pilot-feasibility trial, designed primarily to evaluate procedural feasibility, estimate effect sizes, and inform power calculations for a future definitive RCT rather than to confirm efficacy.

Generally, pilot studies have 10–20 participants, which is in line with the number of participants included in this trial [19]. The study outcomes were interpreted with caution, emphasizing effect sizes and trends rather than *p*-values alone, to reduce overinterpretation of statistical significance in this exploratory context. Future larger-scale studies will be powered to confirm these preliminary findings.

The observed post hoc statistical power, calculated based on the actual difference in isometric quadriceps strength (kg) between the two groups, was 0.73. Although this is slightly below the threshold of 0.80, the main outcome and most secondary outcomes reached statistical significance, suggesting the adequately powered detection of meaningful effects and a relatively low risk of type II error. However, non-significant findings should be interpreted with caution, as the reduced sample size may have limited the ability to detect smaller true effects throughout the 30-day longitudinal intervention period.

### 2.4. Sampling Method

The eligible participants were randomly allocated to 2 groups (experimental: kinesiology taping; control: no taping) using a computer-generated random number sequence produced in Microsoft Excel (version 365). Randomization was completed by an independent researcher to secure allocation concealment and reduce selection bias, and the outcome assessor was blind to group allocation. All participants completed pre- and post-testing following the 30-day repeated taping protocol.

### 2.5. Procedure and Data Collection

The eligible candidates were recruited after passing the inclusion criteria, provided written informed consent, and screened in a clinical interview at the SUSF Championship.

Once screened for eligibility, the participants were randomly allocated to the kinesiology taping or control group. The pre-and post-test assessments were conducted, and the outcomes were compared within and between the groups to evaluate longitudinal changes over the intervention period.

During the 30-day longitudinal intervention, the participants in both these groups continued their regular futsal training schedules (three 90 min training sessions per week and one competitive match every two weeks). Each training session included a standardized structure comprising a 15 min dynamic warm-up, 60 min of technical and tactical drills, and a 15 min small-sided game. All the sessions were conducted in the evening under the supervision of the team coach to ensure consistency across the participants.

To maintain consistency between groups, training adherence was monitored weekly and cross-checked by the researcher during follow-up visits using weekly training logs completed by the athletes and verified by the team coaches. These logs documented attendance and session completion throughout the study period, confirming that both groups maintained comparable training loads. Although individual variations in training effort cannot be entirely eliminated, the use of verified weekly training logs provided reasonable assurance that overall training exposure remained equivalent under real-world conditions.

The training loads were kept consistent between the groups throughout this study period to minimize potential confounding effects and to maintain ecological validity representative of real futsal conditions. All testing was performed on the dominant leg, as identified by ball-kicking preference. The assessments began with isometric and isokinetic quadriceps strength testing, followed by functional performance tests (see Section 2.6).

**Figure 1:**
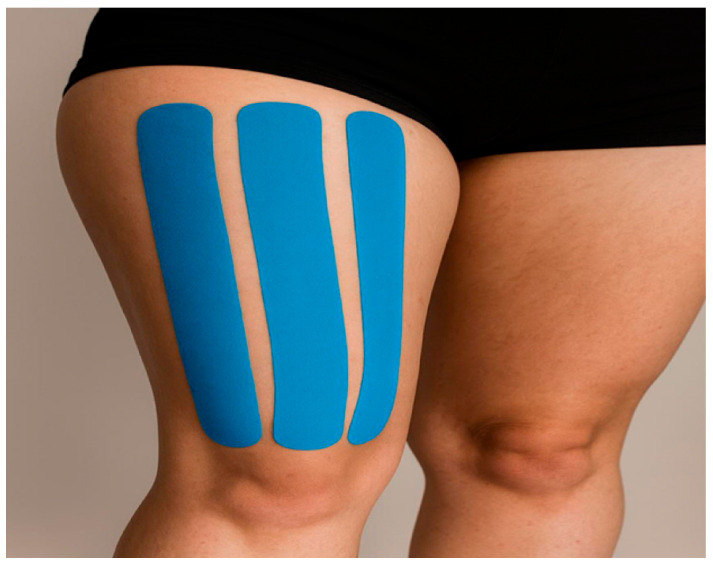
Kinesiotape application.

Kinesiology taping protocol: The taping application described by Kase et al. (2003) was implemented [1] (Figure 1). Both legs were taped during each application to maintain symmetry and ensure consistent neuromuscular stimulation throughout the intervention period.

- Tape characteristics: Kinesio^®^ Tex is a 5 cm width, blue, latex-free elastic therapeutic tape, which is water-resistant and breathable (Kinesio Holding Corporation, Albuquerque, NM, USA).
- Preparation: The skin was shaved and cleaned with alcohol; the tape edges were rounded.
- Application: The tape was applied from the proximal side of the muscle, passing down to the inferior surface of the patella. Three I-strips were applied, one each to the rectus femoris, the vastus medialis, and the vastus lateralis from origin to insertion with ~50% stretch. Anchors were applied without tension and rubbed to improve adhesion following the standard quadriceps facilitation guidelines [20].
- Schedule: Each application was left on for 5 days between applications by the researcher (6 repeated applications over a 30-day period). No washout period was introduced between applications because the aim was to examine the cumulative neuromuscular effects of sustained, repeated kinesiology taping under real-world athletic conditions, rather than isolated short-term responses. This schedule is consistent with the manufacturer’s recommendations (2–7 days of usual wear time). If detachment occurred (e.g., during training or bathing), the same certified researcher reapplied the tape using the identical procedure.
- Safety: The participants were instructed to report irritations. No negative effects were reported.

All taping was performed by the same licensed physical therapists (Saudi Commission for Health Specialties), who had formal training in kinesthetic taping methods. Post-intervention measures were obtained at the end of the 30-day longitudinal protocol.

Three blue I-strips (5 cm width) were applied to the rectus femoris, the vastus medialis, and the vastus lateralis muscles from origin to insertion with approximately 50% stretch, with anchors applied without tension. The tape extended from the proximal thigh to the inferior surface of the patella.

### 2.6. Instruments

Each participant performed a 5 min warm-up session (cyclist with no resistance), followed by 4 sets of 30 s static stretching of the quadriceps muscle. The participants were provided a 30 s rest period between the stretch sets. After this, three validated instruments were used to assess quadriceps strength and lower limb function [17]. All assessments were conducted twice—at baseline and again after completion of the 30-day longitudinal taping protocol—to capture changes over time. The instruments used are listed below.

**Figure 2:**
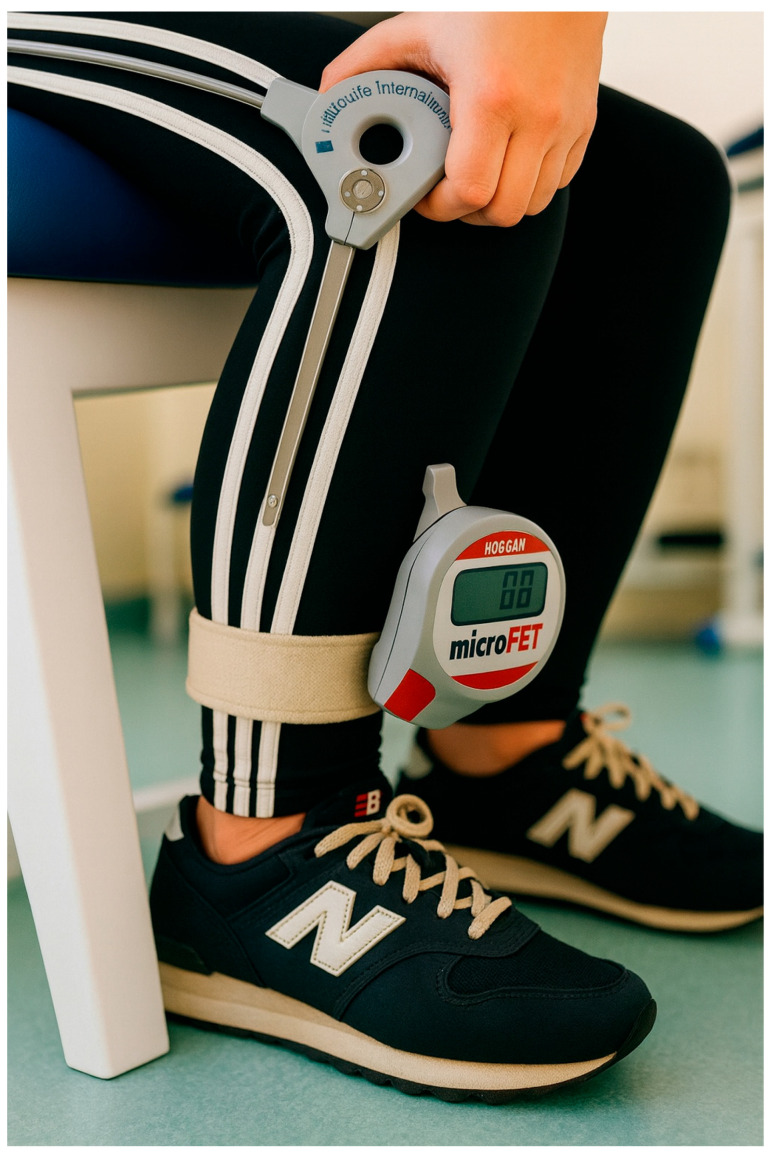
Isometric quadriceps strength test (digital hand-held dynamometer).

1. Isometric quadriceps strength test (digital hand-held dynamometer):

Isometric quadriceps strength was measured using a hand-held dynamometer (microFET®, Hoggan Scientific, LLC, West Jordan, UT, USA) after a 10 min recovery period. During the test, the participants were seated with the hips at 90° flexion and knees at 60° flexion, with the trunk and the hips secured with straps. The dynamometer was positioned on the anterior aspect of the leg, 2.5 cm above the lateral malleolus. The participants crossed their arms in front of the trunk and were instructed to push maximally. After an initial familiarization trial involving submaximal contraction, the participants completed three repetitions involving maximal contractions for five seconds at 30 s of rest in between [21]. This protocol was repeated at post-intervention to evaluate longitudinal changes in isometric performance. The setup for this test is shown in Figure 2.

**Figure 3:**
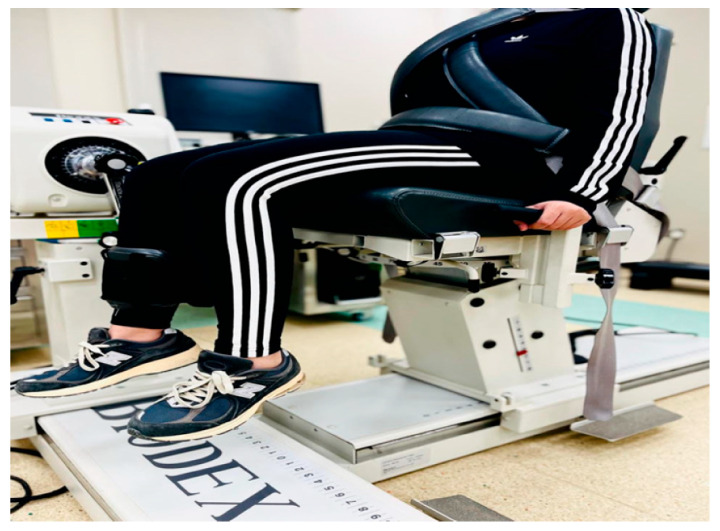
Eccentric/concentric isokinetic strength test (Biodex System).

2. Eccentric/concentric isokinetic strength test (Biodex System):

A total of 10 min of recovery elapsed before performing the eccentric and concentric quadriceps strength testing procedures using the Biodex System (Biodex Medical Systems, Inc., Shirley, NY, USA), which permitted constant resistance and velocity determination over a restricted range of motion. The participants were seated in the Biodex with back support, and the thigh, the ankle, and the trunk were secured with straps, while the knee joint was also secured. Strength testing was completed at angular velocities of 120°/s, 180°/s, and 300°/s, each with 10 repetitions and a 30 s rest between the sets. Torque curves were created, and maximum torque was positioned at the peak of the curve. We identified values for maximum peak torque and power output at the highest peak torque-to-body weight ratio for flexion and extension. This procedure has demonstrated excellent reliability in previous studies, with the intraclass correlation coefficient ranging from 0.82 to 0.95 [22]. This entire Biodex protocol was repeated at post-intervention to compare pre- and post-values and assess longitudinal changes in eccentric and concentric quadriceps performance. The participants’ position during this test is shown in Figure 3.

**Figure 4:**
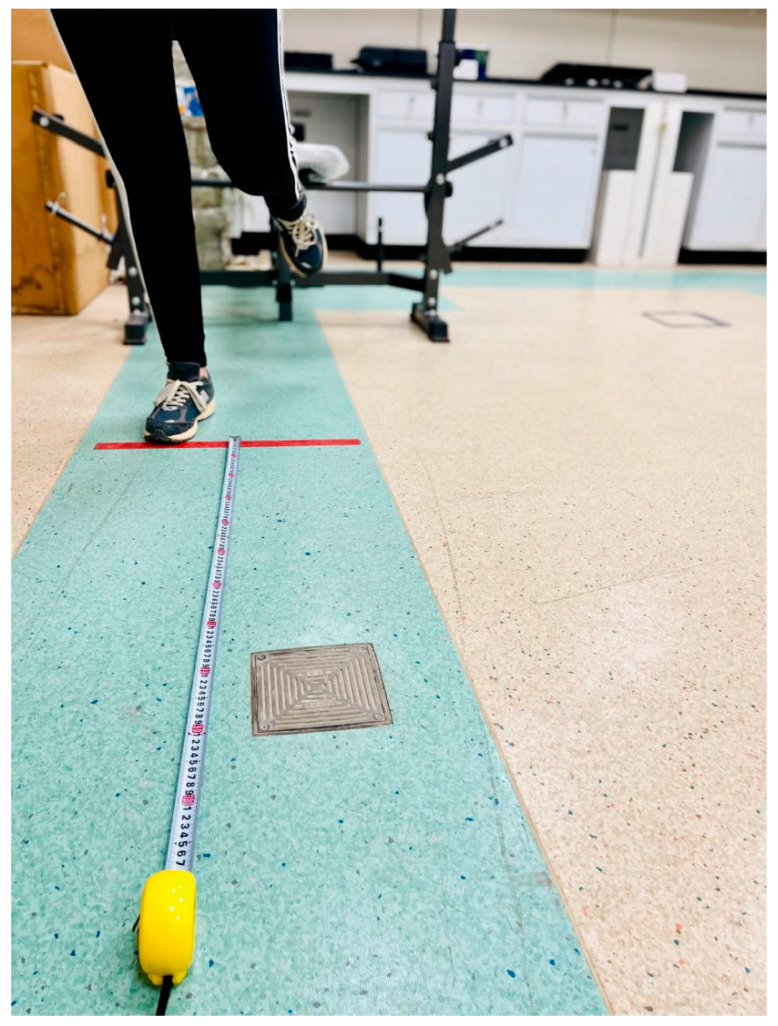
Single-leg hop for distance.

3. Single-leg hop for distance:

To assess functional performance, we utilized the single-leg hop test. The participants started from a standing position on their dominant leg with their arms behind the trunk and were asked to hop forward as far as possible, landing on the same leg and maintaining balance for at least 2 s. Each participant completed a total of 3 trials, and the distance was measured from the big toe at take-off to the big toe at landing, with the average from the 3 trials used in the analysis [23,24,25]. The single-leg-hop test was administered at both baseline and after the 30-day longitudinal taping protocol to determine functional performance changes over time. The single-leg hop for distance test is illustrated in Figure 4.

#### Reporting Guideline Compliance

**Figure 5:**
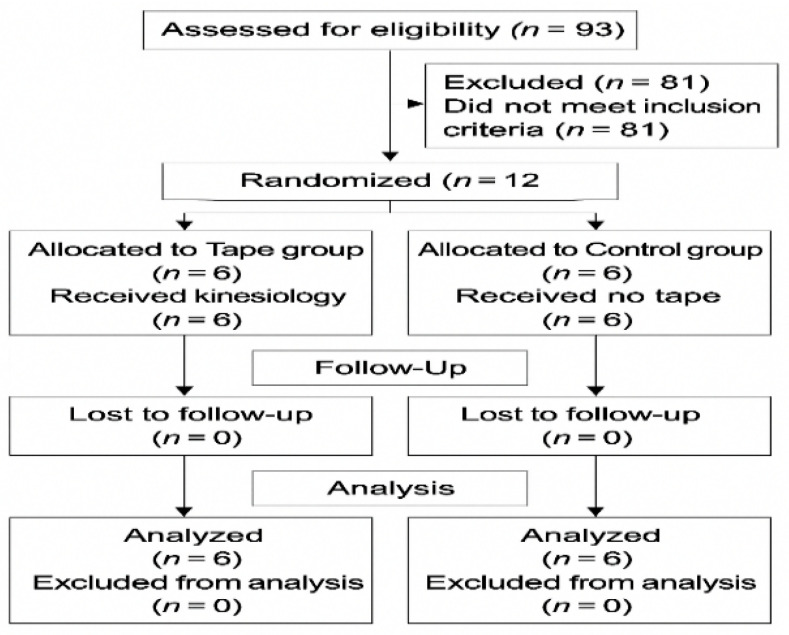
CONSORT flow diagram illustrating participant recruitment, randomization, allocation, follow-up, and analysis procedures in this study.

This study was conducted in accordance with the CONSORT guidelines for randomized controlled trials. A CONSORT flow diagram (Figure 5) illustrates the process of recruitment, randomization, allocation, follow-up, and analysis.

### 2.7. Statistical Data Analysis

All analyses were performed using IBM SPSS Statistics (Version 28). Descriptive statistics (means, standard deviations, and skewness coefficients) summarized participants’ characteristics (age, weight, height, and BMI).

The Shapiro–Wilk test was used to assess normality. Because the small sample size produced some non-normally distributed variables, non-parametric tests were selected as the primary analyses. The Wilcoxon signed-rank test was utilized to compare the outcomes for each group pre-and post-intervention, and the Mann–Whitney U test was used to evaluate the post-intervention differences between the groups. We studied isometric quadriceps and eccentric/concentric dynamic strength and measures of lower limb function to capture within- and between-group longitudinal changes over the 30-day intervention period.

Effect sizes (r) were reported and interpreted as small (0.1), medium (0.3), or large (≥0.5) according to Cohen’s criteria [24].

To complement the non-parametric analyses and explore potential interaction trends over time, a 2 × 2 mixed-model ANOVA (Group: KT vs. Control × Time: Pre vs. Post) was conducted as a supplementary exploratory test. This approach provided a check for consistency of direction and magnitude of effects but was interpreted with caution, given the limited sample size and the violation of normality assumptions. Accordingly, the non-parametric outcomes were prioritized for inferential interpretation.

All tests were two-tailed with significance set at *p* < 0.05. No data were missing; therefore, complete case analyses were performed.

### 2.8. Ethical Considerations

This study was conducted in accordance with the Declaration of Helsinki and was approved by the Institutional Review Board of the College of Medicine, King Saud University, Riyadh, Saudi Arabia (approval code: KSU-HE-23-091; January 2023). All participants provided written informed consent prior to enrollment in January 2023. The trial was registered retrospectively at ClinicalTrials.gov (Identifier: NCT06805227) on 22 January 2025. Because the study began enrollment earlier, we acknowledge that the registration did not occur prior to the first participant’s enrollment.

However, we declare that the study protocol, outcome measures, and data collection schedule were defined before participant enrolment and were not modified thereafter. Future studies will be designed to ensure prospective registration in compliance with ICMJE standards.

All the participants signed a written informed consent form after being given a standardized oral and written briefing on the study purpose, procedures, risks, and potential benefits, and then given the opportunity to ask questions. Participation was voluntary, and they were free to withdraw from this study at any time without penalty. The data collected were anonymized, stored securely, and only accessible by key personnel, which ensured confidentiality and privacy were protected. All the kinesiology taping procedures were conducted by a licensed physical therapist. The participants were continually monitored throughout the trial, and any potential adverse events were recorded so the investigator could be made aware of them; however, no adverse events occurred.

## 3. Results

### 3.1. Baseline Characteristics

A total of 12 people participated and were equally assigned to each group (*n* = 6, control and experimental groups). The age, weight, height, and BMI of the participants in these groups did not significantly differ at the baseline (all *p* > 0.05; Table 1). The skewness coefficients for all the variables were between −1.02 and 0.67, which indicates that the data was approximately normally distributed. These baseline characteristics established comparable starting points for both groups before initiating the 30-day longitudinal taping intervention.

### 3.2. Effects of the Intervention Within and Between Groups at Pre- and Post-Intervention

The groups did not show statistically significant differences in any of the pre-test values (all *p* > 0.05; Table 2), indicating that they were equivalent before the intervention.

In the control group, pre–post comparisons revealed no statistically significant differences for any of the outcome measures (all *p* > 0.05; Table 2), confirming that quadriceps strength and functional outcomes remained stable during the study period without intervention.

In the experimental group, significant pre–post improvements were observed in isometric quadriceps strength, peak torque/body weight during flexion and extension, power output during extension, and lower-limb functional performance (all *p* < 0.05; Table 2). Power output during flexion did not change significantly (*p* > 0.05), although the effect size indicated a moderate improvement. These findings suggest progressive longitudinal gains across repeated kinesiotaping applications.

Post-intervention comparisons demonstrated that the experimental group achieved significantly greater improvements than the control group in isometric quadriceps strength, peak torque/body weight during flexion and extension, and power output during extension (all *p* < 0.05; Table 2). No significant between-group differences were found for power output during flexion or lower-limb function (*p* > 0.05), although moderate effect sizes suggested potential trends favoring the experimental group.

Post hoc pairwise comparisons for all pre- and post-intervention conditions (within- and between-group differences) are summarized in Table 2. These analyses present the detailed Wilcoxon and Mann–Whitney results with effect sizes (*r*) and *p*-values. To explore these findings and evaluate overall interaction effects, 2 × 2 mixed ANOVA analyses (Group × Time) were subsequently conducted, and the results are summarized in Table 3.

### 3.3. Group × Time Interaction Analyses

A 2 × 2 mixed ANOVA (Group: KT vs. Control × Time: Pre vs. Post) was performed as an exploratory supplementary analysis to evaluate the interaction effects. The results revealed significant Group × Time interactions for isometric quadriceps strength (*p* = 0.016), peak torque/body weight during flexion (*p* = 0.021), peak torque/body weight during extension (*p* = 0.014), and power output during extension (*p* = 0.012), all favoring the kinesiotaping group. No significant interactions were found for power output during flexion (*p* = 0.208) or lower-limb function (*p* = 0.117). The significant Group × Time interactions for quadriceps strength, torque, and power outcomes were consistent with the improvements observed in the post hoc pairwise analyses (Table 2). These interaction effects reflect longitudinal improvements in muscle performance across the 30-day intervention period in the kinesiotaping group.

## 4. Discussion

This study examined the impacts of kinesiology taping (KT) on quadriceps strength and lower limb function among female futsal players. The major findings showed significant changes in both the isometric and concentric/eccentric quadriceps strength performances in the KT group, along with some within-group changes to functional performance, after 30 consecutive days of repeated longitudinal application. However, the between-group changes in functional outcomes were not statistically different. These results provide sex- and sport-specific evidence about futsal, an under-represented population at a high risk for quadriceps and knee injuries [4,5,17]. Given the exploratory nature of this pilot study and the small sample size, these results should be interpreted with caution. The limited statistical power increases the potential for both false-positive and false-negative findings, and therefore, the observed effects cannot be considered conclusive. Rather, they serve to inform the design, feasibility, and sample size estimations for future adequately powered randomized controlled trials.

The findings align with the previous studies that reported an increase in quadriceps torque and endurance in fatigued athletes [25] and in clinical populations following stroke or meniscectomy [9,26]. The longitudinal improvements observed in this study suggest that repeated taping sessions may produce cumulative neuromuscular adaptations over time, consistent with the proposed mechanism of KT enhancing sensory feedback and motor-unit recruitment. However, other well-controlled trials of healthy athletes reported no effect of KT on quadriceps torque or activation [27,28]. This difference may be explained by a ceiling effect among non-fatigued, healthy participants, where baseline neuromuscular recruitment is already at the maximum level, and therefore a measurable effect is unlikely to be found [29,30].

It also seems that the level of physical activity may be influential. In our trial, the eccentric and concentric outcomes improved significantly, especially in peak torque/body weight and power output during extension. These outcomes agree with Hung et al. [31], who found improved endurance with KT in athletes, and disagree with Vithoulka et al., who found no changes in untrained adults [32]. There is likely a level of physical activity that modulates the effect of KT because competitive athletes may have an increased sensitivity to proprioception and increased neuromuscular recruitment compared to recreational populations.

In a sports context, KT outcomes may also be influenced. Annino et al. showed improved jump and sprint performances in soccer players [12], while other studies in a soccer context found mixed or no results [10]. Compared to outdoor soccer, futsal has higher demands for high-intensity directional changes and eccentric loading on the quadriceps due to the smaller field and increased speed [33,34]. These demands may increase the neuromuscular benefits of KT, particularly when applied repeatedly over time as in the present longitudinal design, thus making futsal a better context for its application.

Sex differences are another important consideration. Female athletes exhibit a more quadriceps-dominant movement pattern and an increased ACL injury risk compared to males [5]. Takagi et al. directly linked quadriceps weakness among women to increased injury risk [35]. Overall, our finding of improved quadriceps strength among female futsal players indicates that KT may act as a compensatory mechanism to improve neuromuscular control of female athletes, who are at an increased risk of injury, but somewhat contrary to several male studies that reported smaller or null effects [36,37] that may be attributed to sex-specific biomechanical and hormonal differences. Qi et al. emphasized biophysical vulnerabilities that are specific to sex [38]. This highlights the critical importance of generating female-specific data in sports medicine [39].

The longitudinal duration of the intervention is also critical. Our 30-day repeated-application protocol produced consistent improvements, contrasting with short-term studies reporting null results. For example, Korman et al. [37] found no changes in quadriceps activation after one application of KT and Halski found similar results in volleyball players. Longer-applied protocols appear to have some benefit. For example, Annino et al. [26] reported improvements in soccer players with several applications of KT, and Ahmed et al. reported torque gains in patients after a meniscectomy with KT as a routine protocol. Overall, these findings collectively suggest that the therapeutic effects of KT are cumulative and time-dependent, requiring longitudinal exposure for optimal neuromuscular facilitation.

The type of tape and comparator used may also explain these discrepancies. KT is not rigid or non-elastic, as it still allows for active movement, while providing continued stimulation to the cutaneous system to provide extra proprioception and motor recruitment [40,41]. While sham taping comparators may underestimate the cumulative effects according to tension and anatomical arrangements, Tran et al. did not measure these differences following application [42], even though the anatomical application was biomechanically informed. Similarly, rigid or non-elastic tapes, while useful for joint stabilization, restrict natural movement patterns, potentially limiting athletic performance [40]. Other papers on the subject support the current findings. For example, Annino et al. found an acutely improved performance in soccer players following KT use, while similarly tested individuals using placebo strips did not [12]. Ahmed et al. [26] demonstrated that they could increase quadriceps torque following the application of structured KT following meniscectomy, but this did not occur with an application associated with conventional bandaging. Taken together, these longitudinal findings reinforce that KT’s elasticity and extended-wear capacity produce progressive neuromechanical adaptations that distinguish it from short-term or rigid-tape applications.

The method of application also influenced the results. Our study implemented established guidelines to facilitate the quadriceps muscle group using approximately 50% tension by stretching three I-strips, which has previously been shown to elicit greater torque production. Our results align with Choi and Lee’s study which demonstrated directional quadriceps facilitation provided larger improvements than taping with no specific directional facilitation in fatigued athletes [6]. Dolphin et al. also found beneficial outcomes in quadriceps function when tape was applied correctly and in alignment [43]. Alternatively, trial studies utilizing a generalized or minimal-tension method of taping reported to prevent recurrence, such as Halski et al., reported no significant outcomes [10]. These variations in outcome imply there is a neuromechanical response due to non-specific placement of tape, not applying enough tension, or not maintaining repeated longitudinal applications over time.

The measurement methodology may also account for the discrepancies across these studies. Our results are consistent with some trials using direct dynamometry or isokinetic testing, such as Choi et al. and Son et al., who observed increases in quadriceps torque after fatigued athletes used KT [6,25]. By contrast, other studies reported no significant changes in strength, particularly when alternative methods were used. For example, Korman et al. found no differences in muscle performance when evaluating EMG amplitude or general functional tasks [37]. We used validated dynamometry and isokinetic testing, which directly quantify torque and power. This likely enhanced our ability to detect improvements and may partly explain why positive results emerged where others did not. Consistent use of standardized, reliable measures is essential for advancing the evidence on KT’s effectiveness.

Regarding the performance outcomes, functional improvements were observed within the groups only. The KT group significantly increased their single-leg hop distance, but the between-group difference was not significant, supporting the evidence that sport training plays a strong role in functional performance. KT may act more as a muscle-level facilitator than an independent determinant of gross motor function. It should also be noted that the single-leg hop for distance, while a valid and widely used measure of lower limb function, may not fully capture the multidirectional and agility-based demands of futsal, such as rapid cutting, deceleration, and changes in direction. The previous reports suggest that KT helps to promote proprioception and perceived stability [7,15,20], which may be related to psychological comfort and confidence, even with small or negligible changes in objective measures. Context-specific factors, including training volume and psychological expectancy, and duration of longitudinal exposure, likely contribute to variability in results across studies [12,31].

There are multiple factors that contribute to KT’s effects. Biomechanically, elastic recoil elevates the skin, taking pressure off the mechanoreceptors, creating more interstitial space, and increasing blood flow [1,2,20]. Physiologically, continuous cutaneous stimulation increases the afferent input, proprioception, and motor-unit recruitment [6]. Psychologically, KT may enhance perceived stability and confidence [7,15]. Over repeated longitudinal applications, these combined mechanisms appear to generate cumulative neuromechanical adaptations, helping to explain how KT improves athletes’ performance and readiness in high-demand sports such as futsal. When applied carefully and in an evidence-informed manner, KT should be considered by coaches and clinicians as part of the training and rehabilitation continuum. KT represents a low-cost, non-invasive intervention with the potential to enhance neuromuscular performance in sports that place high loads on the quadriceps. Future research should further investigate long-term outcomes, sex differences, and comparative effectiveness with other supportive interventions.

The randomized controlled study design, the blinded outcome assessment, and the use of validated, objective measures of quadriceps strength and function represent the strengths of this study. The consideration of female futsal players is an original contribution to the literature and helps to address an area of both sex- and sport-specific KT research that needs attention.

This study has several limitations that should be acknowledged. The small sample size (*n* = 12) inherently limits the generalizability of the findings and increases the potential for both false-positive and false-negative results. Therefore, the findings should be interpreted with caution. Nevertheless, this was an exploratory pilot trial designed primarily to assess feasibility, estimate effect sizes, and guide the design of future large-scale randomized controlled trials, rather than to provide conclusive evidence of efficacy.

A no-tape control group was used instead of a sham-taping control. We fully acknowledge that for interventions involving cutaneous sensory input, a sham- or placebo-taping control (e.g., tape applied with no therapeutic tension) would better isolate expectation and sensory effects. This consideration will be addressed in future, adequately powered double-blind randomized trials.

Additionally, the study was conducted over an extended period and included athletes from multiple provinces, making it challenging to fully standardize training loads despite consistent procedures, researcher supervision, and training logs implemented to minimize variability. Finally, only one functional performance test (single-leg hop for distance) was used. While valid and reliable, it may not fully capture the multidirectional agility and deceleration demands specific to futsal. Future studies should include a larger sample, a sham-controlled design, and a broader, sport-specific functional test battery to confirm and expand upon these preliminary findings.

## 5. Conclusions

This longitudinal pilot study demonstrated that repeated kinesiology-taping (KT) applications over a 30-day period significantly improved quadriceps strength among female futsal players, with consistent within-group improvements across isometric, eccentric, and concentric measures, as well as modest functional gains. The longitudinal design of this trial suggests that the neuromuscular effects of KT may be cumulative and time-dependent, emphasizing the value of sustained application protocols.

## Figures and Tables

**Table 1:** The baseline characteristics of the participants in the control and experimental groups (*n* = 6).

Variable	Control Group (M ± SD)	Experimental Group (M ± SD) *	Skewness Coefficient **	*p* Value
Age, years	20.67 ± 0.82	21.83 ± 1.47	0.67	0.13
Weight, kg	54.83 ± 6.43	51.83 ± 2.78	0.39	0.33
Height, m	1.61 ± 0.04	1.59 ± 0.05	−1.02	0.46
Body Mass Index (BMI), kg/m^2^	20.97 ± 1.86	20.36 ± 1.47	0.41	0.54

* Values are expressed as mean ± standard deviation (M ± SD). ** Skewness coefficients within ±1.0 indicate approximate normal distribution.

**Table 2:** Comparison of pre- and post-intervention results within and between the control and experimental groups (*n* = 6).

Variable	Group	Pre-Intervention (M ± SD)	Post-Intervention (M ± SD)	Within-Group Effect Size (r) (*p*-Value)	Between-Group (Pre) Effect Size (r)/*p*-Value	Between-Group (Post) Effect Size (r)/*p*-Value
Isometric quadriceps strength (kg)	Control	95.2 ± 35.8	96.5 ± 37.1	−0.15 (0.61)	−0.25 (0.38)	0.83 (0.01 *)
	KT	92.56± 36.76	161.67 ± 42.09	0.90 (0.03 *)		
Peak torque/body weight (flexion, N·s/kg)	Control	65.8 ± 3.1	65.9 ± 3.0	−0.12 (0.67)	−0.28 (0.33)	0.67 (0.02 *)
	KT	66.0 ± 3.24	68.68 ± 2.37	0.90 (0.03 *)		
Power output (flexion, W)	Control	55.6 ± 6.9	55.8 ± 7.1	−0.09 (0.75)	−0.46 (0.11)	0.55 (0.06)
	KT	55.59 ± 7.13	57.8 ± 1.82	0.38 (0.34)		
Peak torque/body weight (extension, N·s/kg)	Control	161.9 ± 8.6	162.1 ± 8.8	−0.10 (0.72)	−0.58 (0.10)	0.74 (0.01 *)
	KT	162.78 ± 8.53	165.5 ± 10.56	0.90 (0.03 *)		
Power output (extension, W)	Control	116.2 ± 11.1	116.5 ± 11.2	−0.12 (0.69)	−0.55 (0.06)	0.74 (0.01 *)
	KT	116.19 ± 11.13	130.69 ± 9.18	0.90 (0.03 *)		
Lower-limb function (cm)	Control	137.2 ± 14.4	137.5 ± 14.6	−0.11 (0.71)	−0.28 (0.33)	0.37 (0.20)
	KT	137.17 ± 14.38	146.27 ± 15.66	0.90 (0.03 *)		

* Values are presented as mean ± SD. Within-group comparisons (Pre vs. Post) were analyzed using the Wilcoxon signed-rank test, and between-group comparisons (Control vs. KT) were analyzed using the Mann–Whitney U test. A 2 × 2 mixed ANOVA (Group × Time) served as the primary analysis to verify interaction effects and minimize Type I error. Post hoc pairwise results are shown above. *p* < 0.05 indicates statistical significance.

**Table 3:** The 2 × 2 Mixed ANOVA results.

Variable	Main Effect of Time (*p*-Value)	Main Effect of Group (*p*-Value)	Interaction (Time × Group) (*p*-Value)	Simple Main Effect (Group Differences Pre vs. Post)
Isometric Quadriceps Strength (kg)	0.043 *	0.039 *	0.016 *	Pre: ns (*p* = 0.38); Post: *p* = 0.01 * (Experimental > Control)
Peak Torque/Body Weight (Flexion, N·s/kg)	0.047 *	0.041 *	0.021 *	Pre: ns (*p* = 0.33); Post: *p* = 0.02 * (Control > Experimental)
Power Output (Flexion, W)	0.402	0.081	0.208	— (No significant interaction)
Peak Torque/Body Weight (Extension, N·s/kg)	0.036 *	0.033 *	0.014 *	Pre: ns (*p* = 0.10); Post: *p* = 0.01 * (Control > Experimental)
Power Output (Extension, W)	0.031 *	0.028 *	0.012 *	Pre: ns (*p* = 0.06); Post: *p* = 0.01 * (Experimental > Control)
Lower Limb Function (cm)	0.057	0.052	0.117	— (No significant interaction)

* Statistically significant at *p* < 0.05.

## Data Availability

The data used in this study are available upon request from the corresponding author.
